# Early Cretaceous lepidosaur (sphenodontian?) burrows

**DOI:** 10.1038/s41598-023-37385-6

**Published:** 2023-06-23

**Authors:** Ricardo Melchor, Mariano Perez, Pablo Villegas, Nahuel Espinoza, Aldo Umazano, M. Cristina Cardonatto

**Affiliations:** 1 Instituto de Ciencias de la Tierra y Ambientales de La Pampa (Consejo Nacional de Investigaciones Científicas y Técnicas-Universidad Nacional de La Pampa), Mendoza 109, 6300 Santa Rosa, La Pampa Argentina; 2 Departamento de Geología, Facultad de Ciencias Exactas y Naturales, Universidad Nacional de La Pampa, Av. Uruguay 151, 6300 Santa Rosa, La Pampa Argentina

**Keywords:** Geology, Sedimentology, Palaeontology, Palaeoecology

## Abstract

Scarce fossil tetrapod burrows have been recorded in Cretaceous rocks, which is probably linked to the dominant equable climates that existed for most of this period. The occurrence of Cretaceous tetrapod burrows from Patagonia (Chubut Province, Argentina) dated between 118 and 115 million years ago, gives insights into their paleoecology and paleoenvironment. The rocks containing the tetrapod burrows are of pyroclastic origin and represent eolian dunes and ash-fall deposits, some reworked by fluvial currents and others showing soil development. Fossil burrow casts preserved in a paleosol are composed by a ramp with a slightly curved or straight path in plan-view and lacking bifurcation, a rounded termination with no enlargement, showing a reniform cross-section, and are assigned to the ichnospecies *Reniformichnus katikatii.* The strongly flattened cross-sectional shape of the burrow casts and comparison with modern lizard burrows suggest that the producers were lepidosaurs (body mass = 50–323 g). Among Cretaceous fossorial lepidosaurs from Patagonia, the best candidate is an eilenodontine sphenodontian. Sphenodontians burrowed in the fossil soils where also arthropods, earthworms and shrubby plants thrived. The rare occurrence of tetrapod burrows in Cretaceous rocks is linked to stressing conditions related to frequent arrival of volcanic ash and a semiarid seasonal climate.

## Introduction

**Figure 1:**
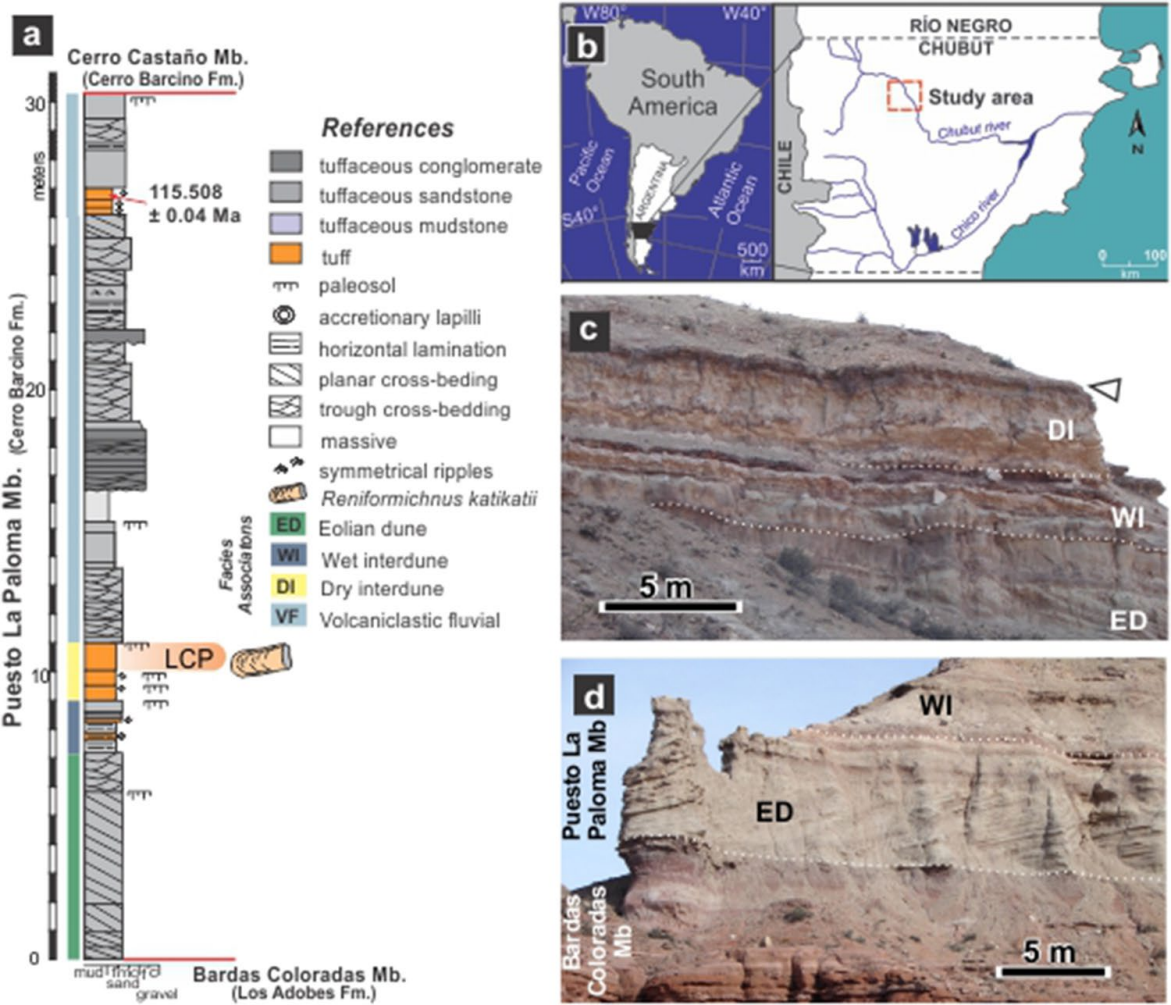
Location, stratigraphic section and exposure of the Puesto La Paloma Member of the Cerro Barcino Formation. (**a**) General stratigraphic section of the Puesto La Paloma Member at the study area, showing the dated level^18^ and the Los Chivos paleosol (LCP) containing the tetrapod fossil burrows. (**b**) Location map. (**c**,**d**) Outcrop view of the intermediate and lowermost section of the Puesto La Paloma Member at the study locality. The arrow in (**c**) indicates the Los Chivos paleosol. Abbreviations in **c** and **d** refer to facies associations. DI: dry interdune. WI: wet interdune. ED: eolian dune. See Table S1 and Fig. S2.

Tetrapods excavate for different purposes, including escaping of dehydration and extreme surface temperatures (thermoregulation), for food storage, breeding, hibernation, to avoid predation and/or to escape fires^1,2^. Although burrowing in other ecoregions occur, an underground cool and humid shelter, with stable temperature is especially critical to survive in arid and semi-arid zones^3^. The scarce record of Cretaceous tetrapod burrows^4^ can be a reflection of the dominant equable climates that existed for most of this period^5,6^. The only Early Cretaceous records of tetrapod burrows are possible mammal or reptile burrows from the Hauterivian of Korea^7^ and putative dinosaur burrows from the Albian of Australia^8^. Published records of Late Cretaceous tetrapod burrows are currently restricted to USA and Brazil, including the first and well-documented ornithopod dinosaur den containing the remains of its producer from the Cenomanian of USA^9,10^. Late Cretaceous examples are purported mammal burrows from the Campanian of USA^11,12^ and two examples from the Maastrichtian of Brazil, one assigned to freshwater turtles^13^ and the remaining to a notosuchian producer^14^. In this context, the finding of Early Cretaceous burrow casts from Patagonia give insights on the paleoecology and paleoenvironment where these structures were excavated, in a period with scarce evidence for tetrapod burrowing. The Los Chivos Hill area is located in the north-western part of Chubut Province, Patagonia, Argentina (Fig. 1b) and contains exposures of the Aptian Puesto La Paloma Member of the Cerro Barcino Formation from the Jurassic-Paleogene Somuncurá-Cañadón Asfalto basin^15,16^ (Fig. S1). At the study area, the Puesto La Paloma Member comprises a ~ 31 m thick sub-horizontal succession (Fig. 1a,c,d) mostly composed of sheet-like tuffaceous sandstones interbedded with scarce tuff, mudstone and breccia^17,18^. It records a pyroclastic-rich non-channelized fluvial environment associated with eolian dunes, ash-fall strata and development of paleosols^17,18^. According to radiometric dates from tuffaceous strata (^206^Pb/^238^U method on zircon), deposition of the Puesto La Paloma Member is largely restricted to the Aptian stage. In particular, the studied tetrapod burrows are bracketed by two ages: 118.497 ± 0.063 Ma and 115.508 ± 0.039 Ma^18^ (Fig. S1). The local sedimentary sequence includes well-sorted cross-bedded tuffaceous sandstone interpreted as transverse eolian dunes developed by dominant winds blowing from the northwest (Fig. S2, Table S1). The rest of the sequence represent ash-fall deposits from a distant western volcanic source, which were reworked by fluvial currents after rains and colonized by plants, with the consequent development of soils (Table S1). Semiarid and seasonal climatic conditions prevailed during deposition of the analyzed sequence. This is inferred from the coexistence of deposits of unconfined streams, alkaline carbonate lakes, and eolian dunes, as well as from poorly developed paleosols having indications of waterlogging and calcification^18–20^. Mean annual precipitation obtained using geochemical data from paleosols yielded estimates of ~ 200–700 mm/year^20^. The Los Chivos paleosol is the topmost and better developed soil were tetrapods burrowed (Fig. 1c).

## Results and discussion

### Tetrapod burrow casts

**Figure 2:**
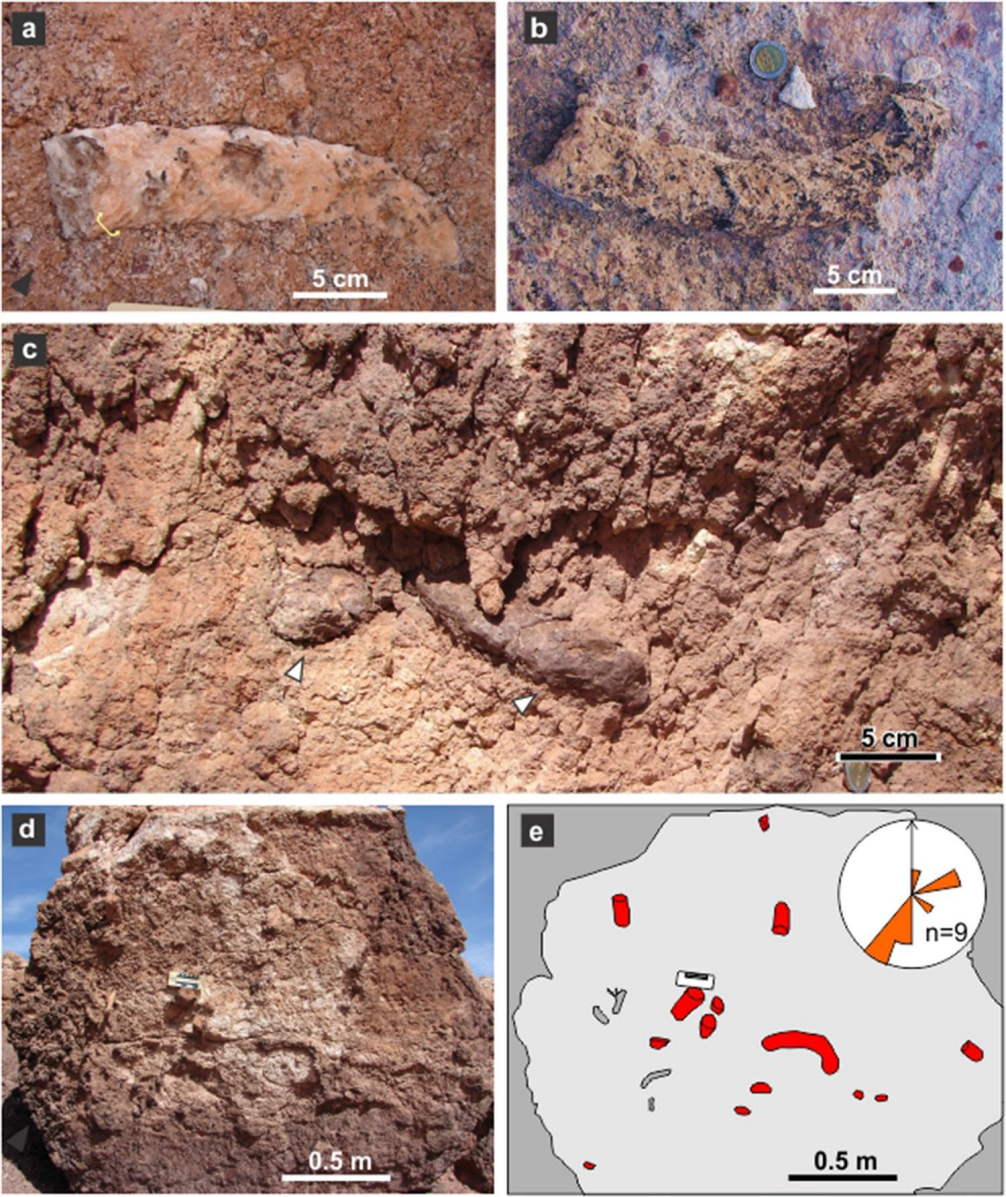
Field occurrence of tetrapod burrow casts. (**a**,**b**) Plan view of curved tetrapod burrow casts. (**c**) Vertical exposure of upper part of the Los Chivos paleosol with two tetrapod burrow casts (arrowed). (**d**,**e**) Plan view of fallen block of top of Los Chivos paleosol with several burrow casts (red) and rhizoliths (gray) and interpretative diagram. The inset in (**b**) is a rose diagram of the dip azimuth of the burrow casts in the block.

**Figure 3:**
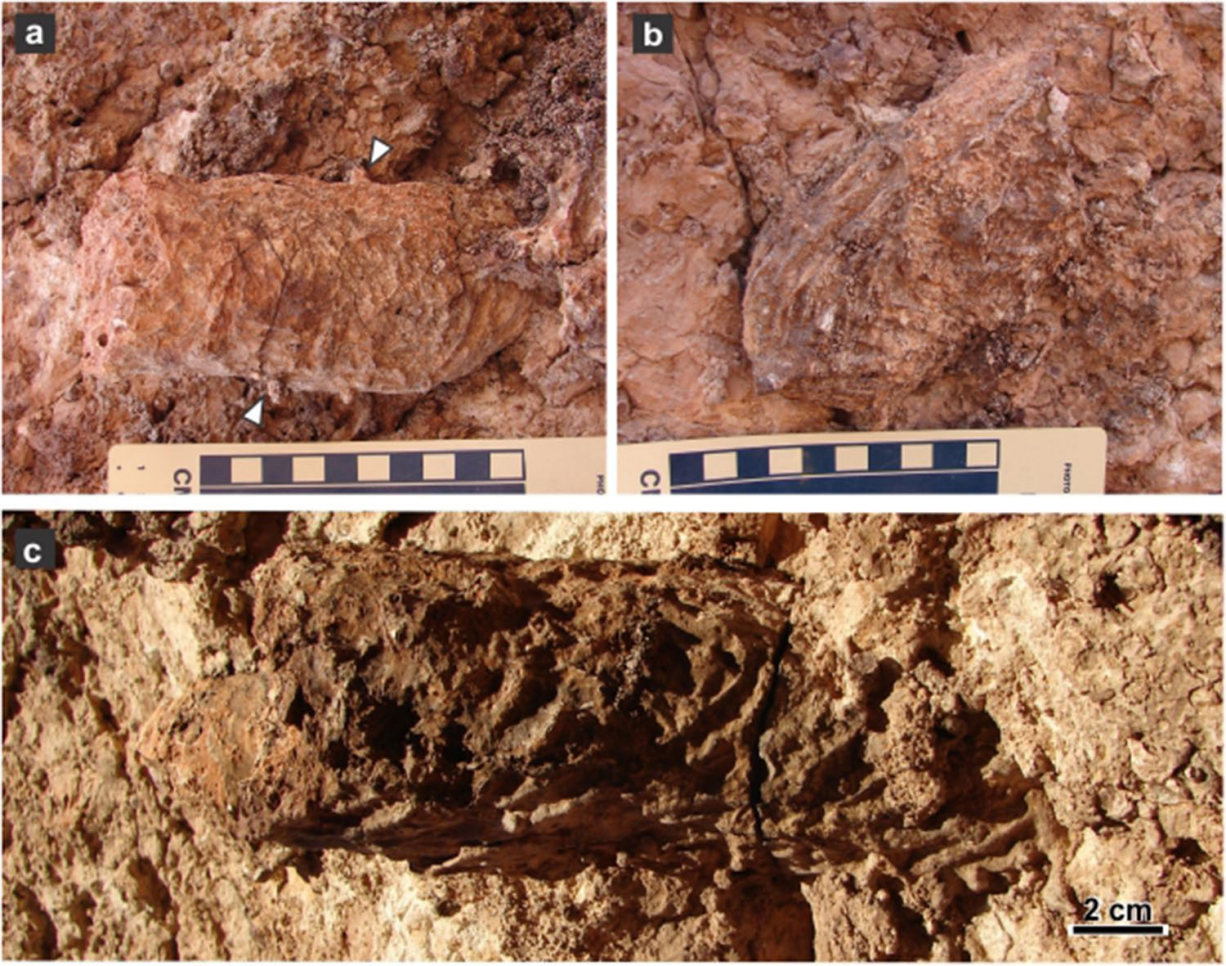
Plan view of tetrapod burrow casts. (**a**,**b**) Rounded and not enlarged terminations (MPEF-IC 4310 and 4312). Arrows in (**a**) points to cylindrical protuberances (invertebrate burrows). Scale divisions are 1 cm. (**c**) Low dipping ramp (MPEF-IC 4311).

**Figure 4:**
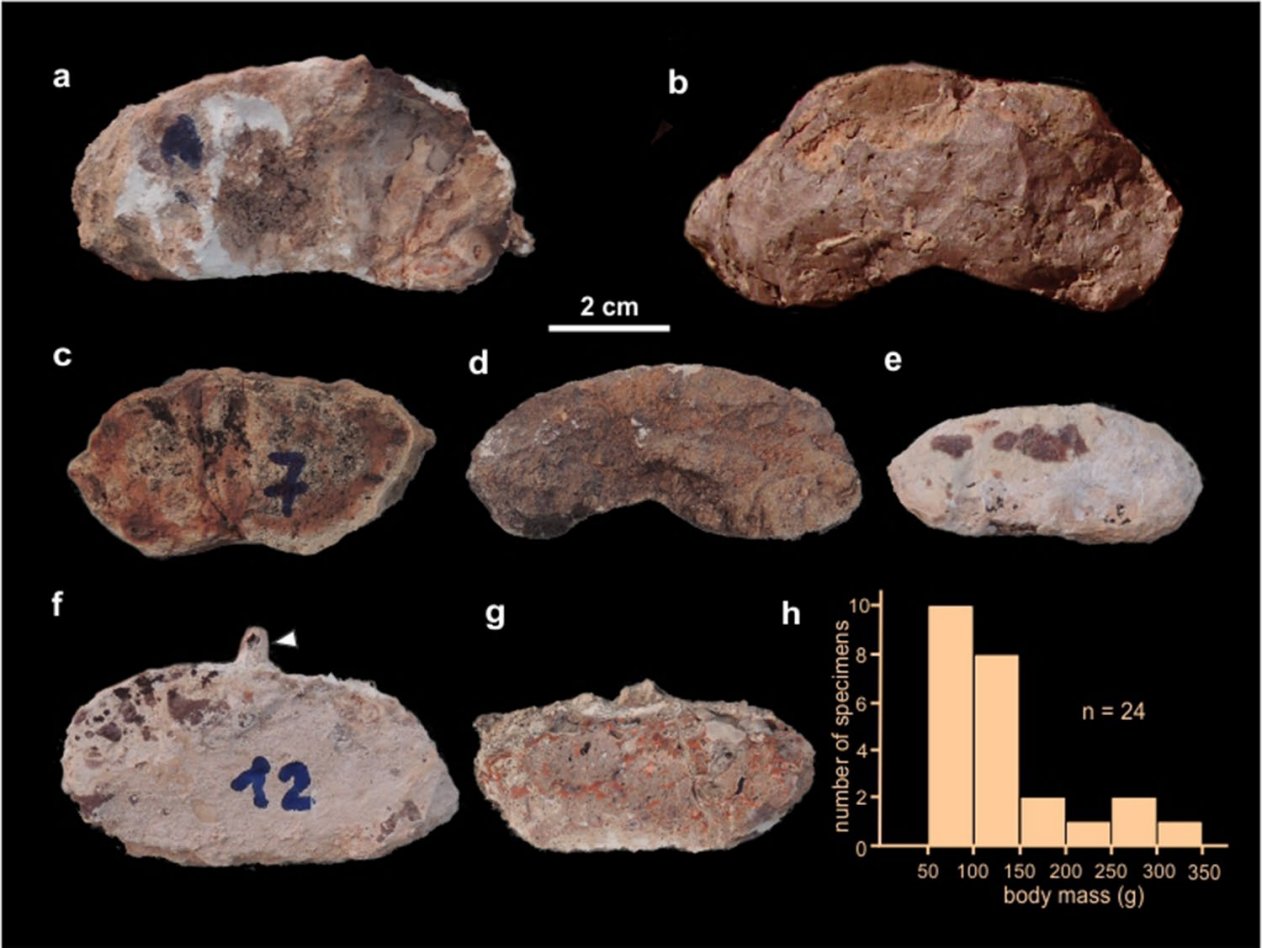
Cross-section shape of *R*. *katikatii* and inferred body mass of the producer. (**a**) MPEF-IC 4310. (**b**) Fs#14. (**c**) MPEF-IC 4314. (**d**) MPEF-IC 4315. (**e**) Fs#11. (**f**) MPEF-IC 4318. The arrow points to an invertebrate burrow. (**g**) MPEF-IC 4312. (**h**) Histogram of inferred body mass obtained using the formula by Wu et al.^32^.

Burrow casts are composed by a ramp (inclined tunnel) with a slightly curved or straight path in plan-view and lacking bifurcation, a rounded termination with no enlargement, and a reniform cross-section (Figs. 2a–c, 3a–c). These morphological attributes allow assignation to the ichnospecies *Reniformichnus katikatii* Krummeck and Bordy^21^ (Table S2 discuss the ichnotaxonomic assignation). Ramp inclination is higher in the proximal end (up to 20°) and tend to subhorizontal at the distal end. Burrow casts occur with apparent orientation and are locally abundant (Fig. 2d,e). Average burrow horizontal diameter is 63.34 ± 2.07 mm, average burrow vertical diameter is 34.26 ± 2.25 mm, and the maximum preserved length is 512 mm (Table 1). Burrow cross-section is consistently elliptical flattened with an average vertical diameter/horizontal diameter ratio of 0.53 ± 0.02. The roof of burrow fills is convex upward and the bottom is commonly bilobed, although some specimens are also convex downward (Fig. 4a–g). In bilobed specimens, the height of the nearly central furrow is up to 20% of the vertical diameter.

**Table 1:** Summary of measurements on collected and field specimens of *Reniformichnus katikatii*.

Specimen #	Dh	Dv	Dv/Dh	Ws	Wt	Ws/Dh
MPEF-IC 4310	63.58	31.77	0.50	8.81 ± 0.41 (7)	1.48 ± 0.05 (21)	0.14
MPEF-IC 4311	67.21	59.09	0.88	12.52 ± 1.25 (5)	1.96 ± 0.10 (14)	0.19
MPEF-IC 4312	69.8	27.32	0.39	9.05 (2)	1.52 ± 0.19 (6)	0.13
MPEF-IC 4313	66.01	37.89	0.57	–	–	–
MPEF-IC 4314	62.44	32.83	0.53	8.94	2.02 ± 0.13 (4)	0.14
MPEF-IC 4315	59.91	30.31	0.51	9.38	1.77 ± 0.28 (3)	0.16
MPEF-IC 4316	47.8	28.15	0.59	8.15 ± 0.91 (3)	1.38 ± 0.06 (11)	0.17
MPEF-IC 4317	55.5	22.75	0.41	4.56	1.19 (2)	0.08
MPEF-IC 4318	62.14	28.79	0.46	9.14 ± 0.66 (4)	1.48 ± 0.07 (14)	0.15
MPEF-IC 4319	60.81	30.4	0.50	–	–	0.19
Fs#1	58.04	20.09	0.35	5.78 ± 0.7 (3)	1.09 ± 0.09 (9)	–
Fs#2	55.85	34.95	0.63	10.08	1.63 ± 0.20 (3)	–
Fs#3	85.39	48.81	0.57	–	–	–
Fs#4	80.26	47.38	0.59	–	–	–
Fs#5	69.81	–	–	–	–	–
Fs#6	62.35	31.04	0.50	–	–	0.13
Fs#7	70.64	36.15	0.51	–	1.00 (3)	–
Fs#8	56	–	–	9.7	1.29 ± 0.23 (3)	0.17
Fs#9	54.16	–	–	8.59	1.36 ± 0.11 (3)	0.16
Fs#10	47.89	21.87	0.46	–	–	–
Fs#11	57.31	35.39	0.62	–	–	–
Fs#12	54.54	–	–	–	–	–
Fs#13	51.61	–	–	–	–	–
Fs#14	60.66	30.65	0.51	–	–	–
Fs#15	76.82	–	–	–	–	–
Fs#16	90.2	49.5	0.55	–	–	–
Fs#17	60.66	30.65	0.51	–	–	–
Fs#18	76.82	–	–	–	–	–
Average ± SE (n)	63.34 ± 2.07 (27)	34.26 ± 2.25 (20)	0.53 ± 0.02 (20)	9.23 ± 2.28 (30)	1.34 ± 0.05 (120)	0.15 ± 0.008 (12)

*Dh* horizontal diameter, *Dv* vertical diameter, *Ws* width of sets of claw traces, *Wt* claw trace width, *Fs* field specimen. Number of readings are indicated between parentheses.

**Figure 5:**
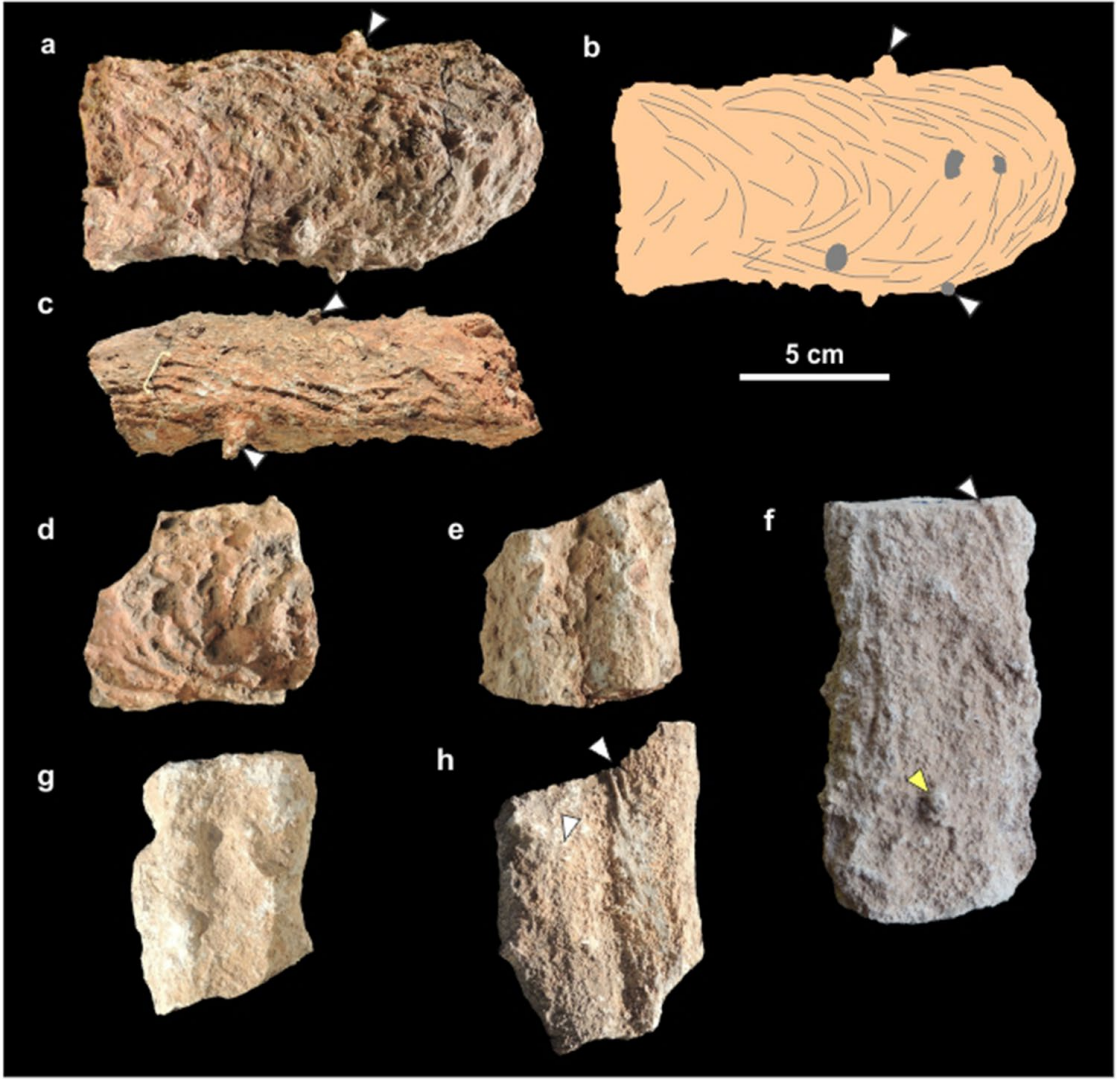
Surface ornamentation of *R*. *katikatii*. (**a**–**c**) Plan view, interpretative diagram and oblique lateral view of MPEF-IC 4310. White arrows and grey areas (in **b**) indicate invertebrate burrows. Bracket in (**c**) indicates a set of claw traces. (**d**,**e**) Claw traces on roof and smooth bilobed bottom in MPEF-IC 4314. (**f**) Nearly flat bottom with subtle claw traces (white arrow) and invertebrate burrow (yellow arrow) in MPEF-IC 4318. (**g**) Smooth bilobed bottom in MPEF-IC 4317. (**h**) Bilobed bottom with claw traces forming a chevron pattern (arrows) in MPEF-IC 4315.

**Figure 6:**
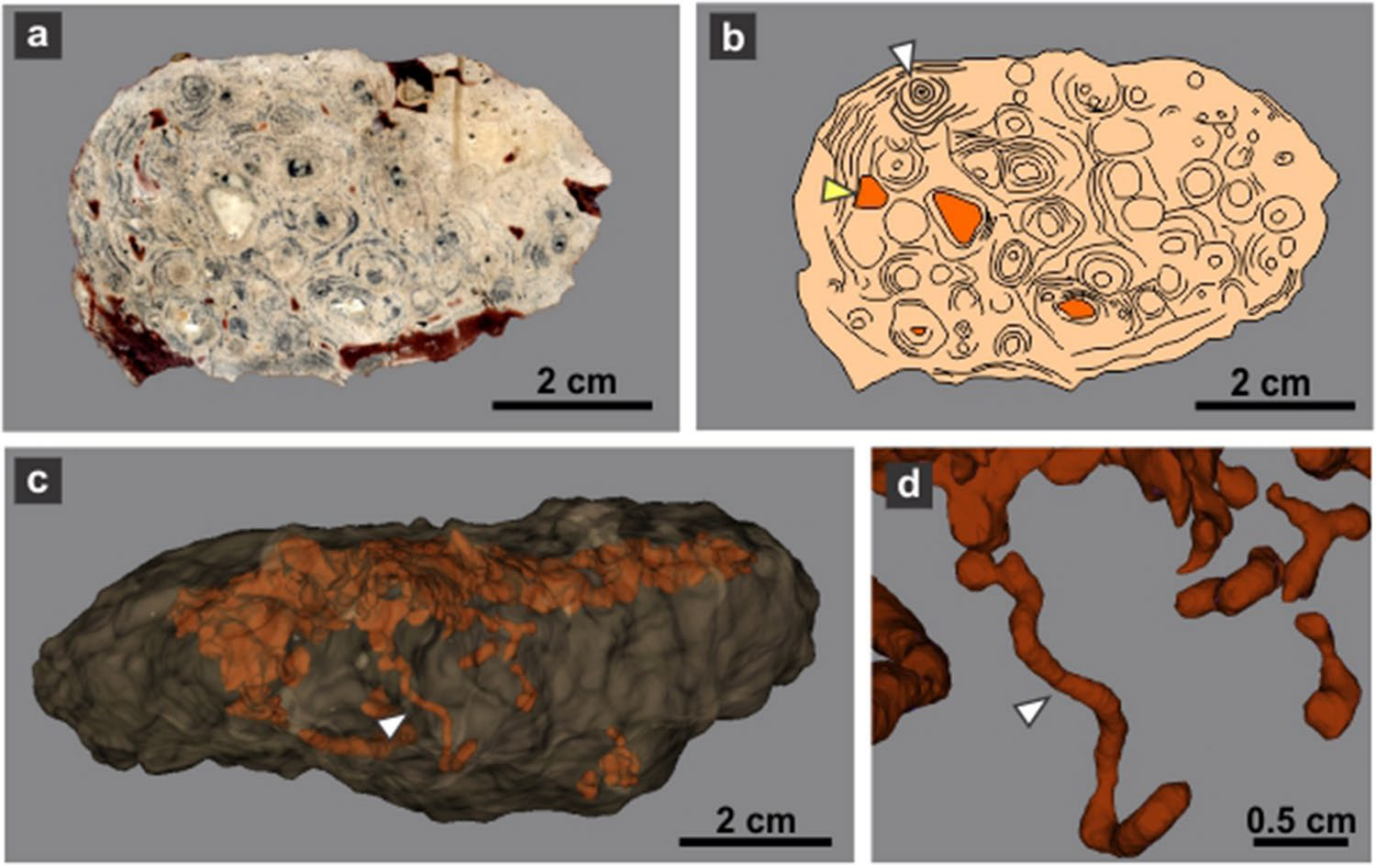
Massive fill of *R*. *katikatii*. (**a**,**b**) Polished cross-section of burrow fill and interpretative drawing. Note accretionary lapilli (white arrow) and pumice clasts (yellow arrow) both surrounded by concretionary cementation. (**c**) Tridimensional model from CT of MPEF-IC 4312 (oblique lateral view) with transparent outline and orange bodies interpreted as denser parts product of cementation. The arrow indicates the subvertical burrow of (**d**). (**d**) Detail of quasi-spiral submillimetric burrow with a rounded and enlarged end.

Most burrow casts display groups of three parallel millimeter-thick ridges that are interpreted as claw trace sets (Figs. 3a–c, 5a–d). Claw traces are sharper and better developed in the roof and lateral side of the burrow casts, where the average width of the sets of claw traces is 8.89 ± 0.55 mm. The pattern of claw traces in the roof is typically arcuate, with the sets of traces starting at the midline and converging in the lateral surface of the burrow cast (Fig. 5a,b). In the bottom, the sets of claw traces average 8.48 ± 0.47 mm in width and compose a low angle to chevron pattern (Fig. 5f,h). Individual claw traces display a consistent width in roof and bottom averaging 1.34 ± 0.05 mm (Table 1). The contrasting claw trace pattern in roof and bottom are tentatively linked to scratch-digging with the forelimbs (arcuate traces in roof) and pushing back loose sediment with the hindlimbs (chevron traces in bottom), respectively. Sparse subcircular and smaller cylindrical burrows having a diameter ranging from 4.4 to 7.5 mm cross-cut the tetrapod burrows (Fig. 5a–c,f). The fill of tetrapod burrows is massive cemented volcanic ash with abundant subspherical concentric structures interpreted as ash aggregates (accretionary lapilli) and vesiculated ash (pumice) clasts, both with concretionary growth (Figs. 4a–h, 6a,b). Computed tomography images of *R*. *katikatii* allow to identify vertical millimeter-thick burrows with rounded and enlarged terminus that postdate tetrapod burrow abandonment and filling by sediments (Fig. 6c,d). Presence of a bilobed bottom is a feature typical of some fossil and extant tetrapod burrows^21–27^. This feature was interpreted as reflecting protracted occupation of a burrow and repeated passage of the occupant, thus producing a differential compaction of the sides of burrow bottom^27,28^.

The burrows were left open by the occupant, as suggested by the massive fill, and passively received input of volcanic ash, either by settling from ash clouds or reworked by currents. The former origin is favored by the presence of accretionary lapilli, that is typically formed subaerially and commonly cannot be reworked^29^. The average ratio between the average width of the sets of claw traces (a proxy for the autopodium size as each trace can be linked to the middle three digits) and the horizontal diameter (an indication of the producer size) is 0.15 (Table 1), which is indicative of a tetrapod origin^30^. The presence of sets of claw traces is suggestive of scratch-digging mechanism, which is employed by a large variety of limbed tetrapods^31^. Using the allometric relationship with the cross-sectional area of burrow casts^32^, the body mass of the producer was estimated as ranging between 50 and 323 g (Fig. 4h).

### Sphenodontians as burrow producers

**Figure 7:**
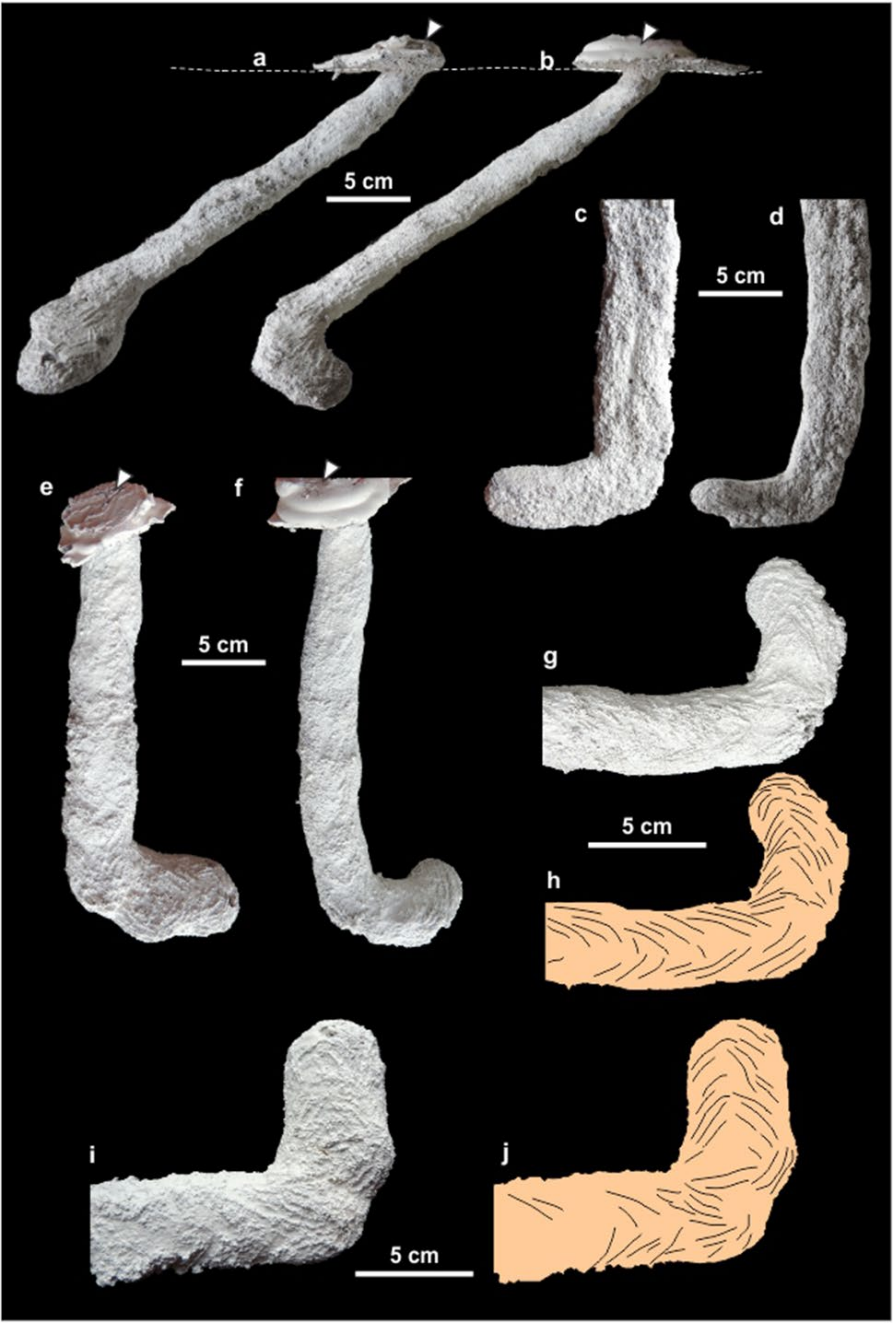
Modern *Liolaemus* sp. plaster burrow casts (**a**,**c**,**e**,**i**, and j belong to GHUNLPam 29090; and **b**,**d**,**f**–**h** to GHUNLPam 29091). (**a**,**b**) Side view. The dashed line marks the terrain surface (see also Fig. S3). Arrows indicate the excess plaster poured in the surface to mark the ground surface. (**c**,**d**) Bilobed bottom, note that the distal end is smooth. (**e**,**f**) Plan view, note distal curvature. Arrows indicate the excess plaster. (**g**–**j**) Surface ornamentation and interpretative drawing of the distal part of burrow casts.

The burrow casts exhibit a diameter, cross-section shape, overall architecture, including the low vertical diameter/horizontal diameter ratio, that are indicative of a producer with a sprawling posture as typical of lepidosaurs^33^. Extant *Liolaemus* sp. (Squamata: Liolaemidae) burrows from central Argentina (Fig. S3) share with the studied tetrapod fossil burrows overall architecture, cross sectional shape (average vertical diameter/horizontal diameter ratio = 0.56 ± 0.01), bilobed bottom and surface ornamentation. Casted *Liolaemus* burrows consists of a simple ramp with a constant horizontal and vertical diameter, having a rounded end without enlargement, with an “L” shaped outline in plan view (Fig. 7a,b,e,f). The bottom of *Liolaemus* casts is bilobed (Fig. 7c,d) as observed in the fossil example, probably indicating a protracted usage of the burrow. The surface ornamentation displays an arcuate pattern (Fig. 7g–j) that is strongly similar to that exhibited by the fossil tetrapod casts (Fig. 5a,b), suggesting a similar excavation mechanism.

Considering the Cretaceous tetrapod fossil record of South America, the candidate for producer of *R*. *katikatii* are lepidosaurs or, less likely, basal mammals. Some basal mammals displayed a sprawling posture, in some cases related to arboreal habits^34,35^, which can be thus discarded. Most of the remains of Cretaceous mammals in South America correspond to cranial fragments and isolated teeth^36–38^, for which is difficult to estimate their body mass (Table S3). A single well-documented mammal taxon is *Vincelestes nuequenianus* (Mammalia, Cladotheria) from Barremian-Aptian deposits of Patagonia, but it can be discarded as producer because of larger size (body mass ranging from 619 to 1228 g) and an inferred scansorial-arboreal habit^39,40^. The Cenomanian taxon *Cronopio dentiacutus* (Mammalia, Dryolestidae) is diminutive (skull width about 10 mm) and is only known from an incomplete skull^41^. A mammalian digger for *R*. *katikatii* can be dismissed considering the cross-sectional shape of the burrows (height/width ~ 0.5) and that the Cretaceous fossil record from Patagonia lacks a candidate with fossorial habits that match the inferred body mass of the producer.

Cretaceous lepidosaurs from South America belong mostly to Squamata in the north and southeast of Brazil, while Sphenodontia is restricted to more southern latitudes, in the south of Brazil and Argentina^42^. The record of Mesozoic Squamata in Patagonia is incomplete and discontinuous, restricted to the Late Cretaceous and mostly composed by unidentified Iguania and Scincomorpha (Table S3). Snakes are not considered potential producers because of their large size and lack of appendages or only presence of vestigial posterior appendages^43–46^. Squamata records are from the Cenomanian–Turonian to early Campanian and belong to fragmentary cranial remains from diminutive individuals (Table S3), which are considered much smaller than the presumed producer. Sphenodontians are known in Patagonia from the Early Cretaceous to the Paleocene (Table S3), most belong to eilenodontines^47–49^ that were gregarious and herbivorous reptiles that lived in burrows excavated using their powerful beaks and hoofed claws^48^, and there is a single small-sized sphenodontine that likely preyed on insects and small vertebrates^50^. There are three potential sphenodontian candidates for the producer of the burrow casts. The only Early Cretaceous record is the eilenodontine *Kaikaifilusaurus* (*Priosphenodon*) *minimus* that was recovered from the same formation than the burrow casts^48^ and the skull length of a subadult specimen is 20 mm, with a inferred body mass below the range for the fossil burrows (Table S3). *Kaikaifilusaurus minimus* is characterized by a flat skull, sharp beak that is slightly procumbent in its anterior portion improving both the excavation and cutting of vegetation, along with the well-developed adductor musculature, tall and robust jaw, wide teeth with low crowns. These features suggest fossorial habits^48^, making *K. minimus* a good candidate for producer of the burrow casts. Another candidate of adequate size is the medium sized sphenodontine *Tika giacchinoi* from Cenomanian beds of northern Patagonia, however, there is no positive evidence that might suggest a burrowing behavior^50^. The third candidate is the eilenodontine *Patagosphenos watuku* recovered from Turonian levels that presents a similar bone microstructure to that of the living *Sphenodon* with medullar cavity reduction of long bones^49^, this feature is interpreted as an adaptation for fossoriality in living mammals^51^. Although the recovered remains are fragmentary, the small incomplete dentary (Table S3) indicates a very small individual to be considered the producer of these burrows. The remaining Late Cretaceous records of sphenodontians from Patagonia are considerably larger or younger than the burrow-bearing unit (Table S3). To summarize, a lepidosaurian origin for the producer of *R*. *katikatii* is indicated by the cross-sectional shape and by comparison with modern lepidosaur burrows (*Liolaemus* sp.). Patagonian Cretaceous Squamata are much smaller than the inferred body mass of the producer and are thus not considered a likely tracemaker. Among the Cretaceous sphenodontians from Patagonia, the most likely burrow digger is *K. minimus* (recovered from the same lithostratigraphic unit that the fossil burrows) because of fossorial habits and similar body mass.

The sphenodontians, very diverse and widely distributed during the Mesozoic, nowadays only live in New Zealand, constituting a relict population. *Sphenodon* lives in burrows of various morphologies, from a simple ramp with a terminal nest to complex systems with several entrances and a nest^52,53^. Simple *Sphenodon* ramps are 110 mm to 500 mm long, have an average height of 45 mm and average width of 73 mm^53^. The ratio between height and width of the cross-section of modern *Sphenodon* burrows is 0.61, indicating an elliptical flattened cross section. The size and overall morphology of *Sphenodon* burrows is similar to those described above for Early Cretaceous burrow casts (Table 1).

Considering the known Cretaceous fossorial lepidosaurs from Patagonia, the inferred size of the of the tetrapod remains (although most are incomplete) and its close relationship with the size of its burrows^54^, in addition to the similarity with the tuatara burrows morphometry, it is suggested that the most likely producers of the burrows described here are the sphenodontians, which were abundant in Patagonia during the Cretaceous (Table S3). In particular, an adult *K*. *minimus* could be the best candidate considering that was recovered from the same formation and the skull features suggesting a fossorial habit. These are the first documented fossil sphenodontian burrows.

### Los Chivos paleosol and ichnological expression of soil biota

**Figure 8:**
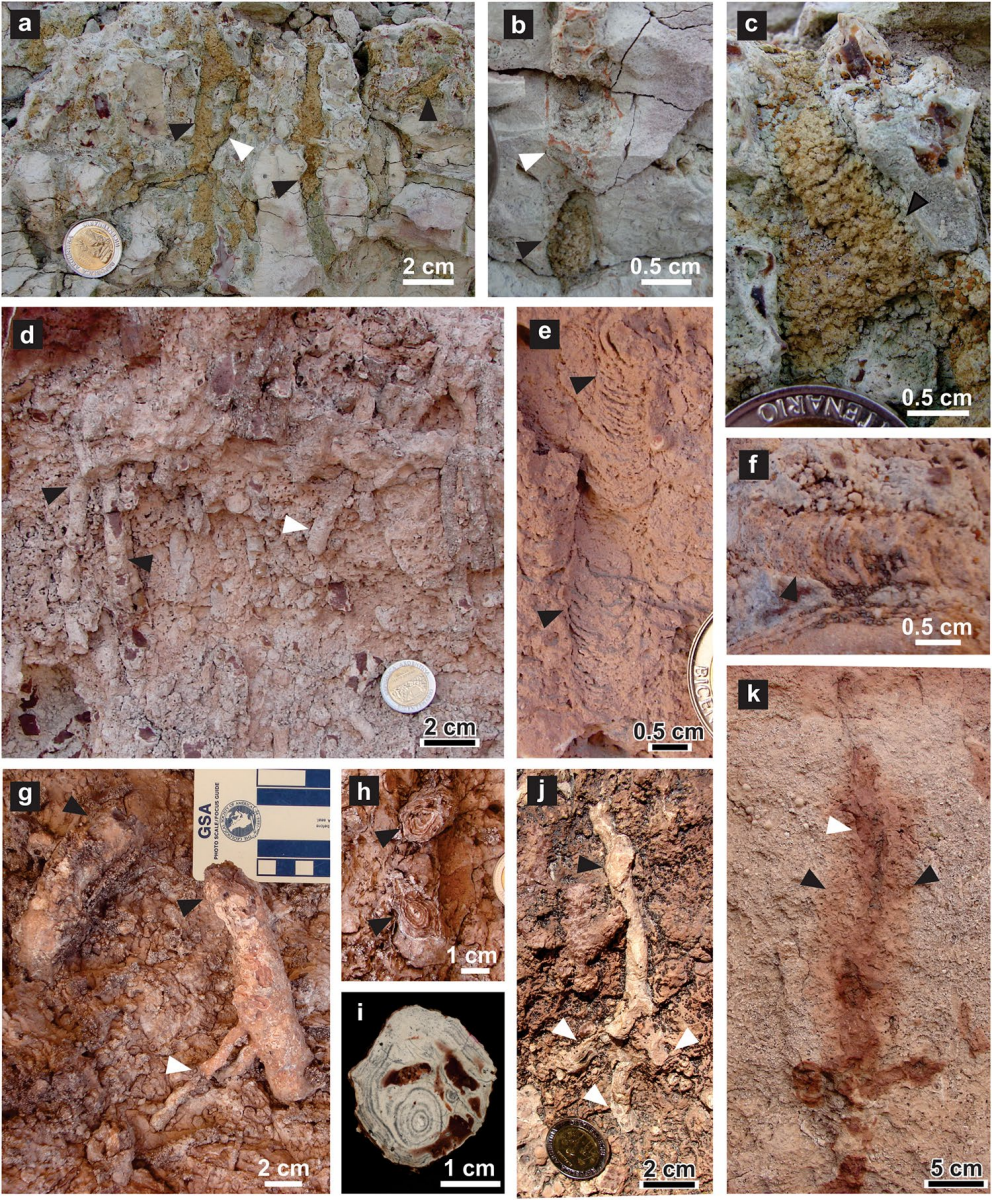
Trace fossils associated with *R*. *katikatii*. (**a**–**c**) *Edaphichnium lumbricatum* composing subvertical burrows (**a**,**b**) and a swelling in (**c**) (black arrows). Note bifurcation (white arrow in a) and meniscate fill (white arrow in **b**). (**d**) *Skolithos linearis* (black arrows) with rounded end (white arrow). (**e**,**f**) *Taenidium barretti* (margin of burrow arrowed). (**g**–**j**) Siliceous rhizocretions (black arrows). Note secondary bifurcation (white arrows in **g** and **j**) and concentric rings (in **h** and **i**). (**g**) and (**h**) are plan views, (**i**) is a polished section and (**j**) is a subvertical exposure. (**k**) Subvertical ferruginous rhizolith, including root cast (white arrow) and lighter halo (black arrows).

The Los Chivos paleosol contains three horizons having transitional boundaries (Fig. S4). The upper horizon is a 0.75 m thick, light pinkish white massive fine-grained tuff with millimetric Fe–Mn nodules. Sphenodontian burrows occur in this horizon, along with vertical meniscate (*Taenidium barretti*) or massive (*Skolithos linearis*) invertebrate burrows and rhizoliths, which are locally abundant. The middle horizon is a 0.60 m thick fine-grained tuff with a coarse granular structure, pinkish white to white in color, showing diffuse parallel lamination. The lower horizon is a 110 cm thick, light grey massive very fine-grained tuff also showing coarse structure (Fig. S4). In addition to the tetrapod burrows, the soil biota is reflected in biogenic structures attributed to earthworms, unidentified arthropods and sparse shrubby plants. Biogenic structures produced by earthworms includes subvertical cylindrical burrows (averaging 7 mm wide) and globose swellings with pelletal filling (ichnospecies *Edaphichnium lumbricatum*) (Fig. 8a–c) occasionally associated with meniscate burrows (ichnospecies *Taenidium barretti*) (Fig. 8e,f). Fecal pellets are yellowish and rounded to elliptical, with an average diameter of 0.87 ± 0.05 mm. *Edaphichnium lumbricatum* has been reported typically in the Cenozoic^55–58^, although also occur in Late Jurassic and Late Cretaceous paleosols^59–61^. Arthropod domiciles are represented by subvertical burrows with massive fill and rounded end (ichnospecies *Skolithos linearis*) that occur profusely in the uppermost part of the Los Chivos paleosol, locally with high density (up to 290 burrows/m^2^) (Fig. 8d). These structures average 8.39 ± 0.22 mm in diameter and can reach 0.10 m in length. *Skolithos linearis* were likely produced by insects or arachnids^62–64^. Evidence about the plant community that thrived in this paleosol is provided by root-generated structures or rhizoliths. Identified rhizoliths are mostly siliceous rhizocretions and ferruginous root casts^65^. Rhizocretions are common and display a concentric internal structure, downward branching and consequent reduction in diameter (Fig. 8g–j). Maximum preserved length is 0.17 m and average diameter is 14.2 ± 1.5 mm (n = 8). Ferruginous rhizoliths are vertical, up to 0.4 m long, with horizontal branching and a central, 20 mm wide, roughly cylindrical, internal brown root cast and a 30–40 mm wide light brown external zone (Fig. 8k). The central tubular zone also displays submillimetric root traces. The size of root structures suggests a sparse shrubby vegetation by comparison with modern analogues^66,67^.

The development of the Los Chivos paleosol occurred under a semiarid and seasonal climate, in a flat area between eolian dunes (interdune) where arrived frequent ash clouds from distant volcanoes, which settled subaerially and were reworked by wind and rainwater (Table S1). A period of prolonged stability (at least 2000–3000 years^68^) with minimum or no arrival of new ash clouds permitted plant rooting and soil development with subsequent establishment of a soil community. The weak alteration degree of the tuffaceous parent material, the moderate distinction of horizons, scarce microscopic features for soil development, and the preservation of the original bedding are characteristics of a moderate to weakly developed paleosol. Consequently, it is best compared with modern andisols or, less probably, andic entisols. The presence of calcite and Fe–Mn oxide coatings suggest a seasonal climate^69^. The frequent arrival of volcanic ash produced barren and xeric landscapes^70^ that probably enhanced the semiarid and seasonal climatic conditions inferred from sedimentary facies and paleosol geochemistry. Although tetrapod burrows are commonly multi-purpose structures, *R*. *katikatii* from the Early Cretaceous of Patagonia was primarily used for shelter to ameliorate seasonal climatic variations and to keep uniform temperature and humidity. We cannot discard the use for breeding and to avoid predation, but there is no evidence supporting food storage, hibernation, or escape from fires.

## Methods

Collected fossil specimens are housed at the Ichnology Collection, Museo Paleontológico Egidio Feruglio (Trelew, Chubut, Argentina) under the acronym MPEF-IC. Plaster burrow casts of extant Squamata are kept at the Paleontological Collection of the Facultad de Ciencias Exactas y Naturales, Universidad Nacional de La Pampa, under the acronym GHUNLPam. Computed tomography (CT) scans of selected burrow casts were carried out at the FAERAC Foundation (Santa Rosa, La Pampa, Argentina) with a medical tomograph Toshiba Aquilon One 320 (MPEF-IC 4310, 4312) and Siemens SOMATOM go.Now (MPEF-IC 4311, 4318). CT scans generate slices in sagittal, coronal and axial views where each type of view has its own spacing ranging from 0.112 to 0.300 mm. The 3D Slicer software^71^ (https://www.slicer.org) was used for the analysis, processing and three-dimensional visualization of the CT scans. Tridimensional photogrammetric models of selected burrow casts were generated based on photographs taken with a Nikon Coolpix L830 camera and processed in the software Agisoft Metashape Pro™. The resulting models were exported in OBJ files to Adobe Photoshop CC™ and converted to U3D files (a standard format for 3D), to compose a PDF file for easier visualization.

Cross-sectional area of fossil burrow casts (Ab, cm^2^) was estimated using scaled photographs of the collected and field material with the software ImageJ (https://imagej.nih.gov). This value was then employed to estimate the body mass (Mb, g) of the producer using the allometric relationship^32^ Ab = 0.46 Mb^0.74^. Body mass of fossil Sphenodontia was estimated using the relationship between head length (HL, mm) and body mass for extant specimens of *Sphenodon punctatus*^72^. The data is herein fitted by equation Mb = 3.3899^0.081HL^ (R^2^ = 0.9106, n = 209).

Micromorphological descriptions of paleosol was conducted with a Nikon Eclipse E400 POL petrographic microscope following standard procedures^73–75^. Burrow cast measurements are expressed as average values ± standard error and indicating the number of measurements.

## Supplementary Information

Supplementary Information.

## Data Availability

The datasets generated and/or analyzed during the current study are available in the Supplementary Material and Figshare.com repository. CT and photogrammetric tridimensional model of specimen MPEF-IC 4310 https://figshare.com/s/ee80e2be44cd2148209c, photogrammetric tridimensional model of specimen MPEF-IC 4311 https://figshare.com/s/00407b3cdef2e38eadb9; photogrammetric tridimensional model of specimen MPEF-IC 4312 https://figshare.com/s/4c9495e49143371181eb.
