# Supplementary material for: Early Cretaceous lepidosaur (sphenodontian?) burrows
Source: Sci Rep. 2023 Jun 23;13:10209. doi: 10.1038/s41598-023-37385-6 (PMC10290101; doi:10.1038/s41598-023-37385-6)
Supplement: Supplementary file 1 — Supplementary Information. [file 41598_2023_37385_MOESM1_ESM.pdf]

Supplementary Information

Aptian Lepidosaur (Sphenodontian?) burrows

Ricardo Melchor, Mariano Perez, Pablo Villegas, Nahuel Espinoza, Aldo Umazano, and M. Cristina Cardonatto

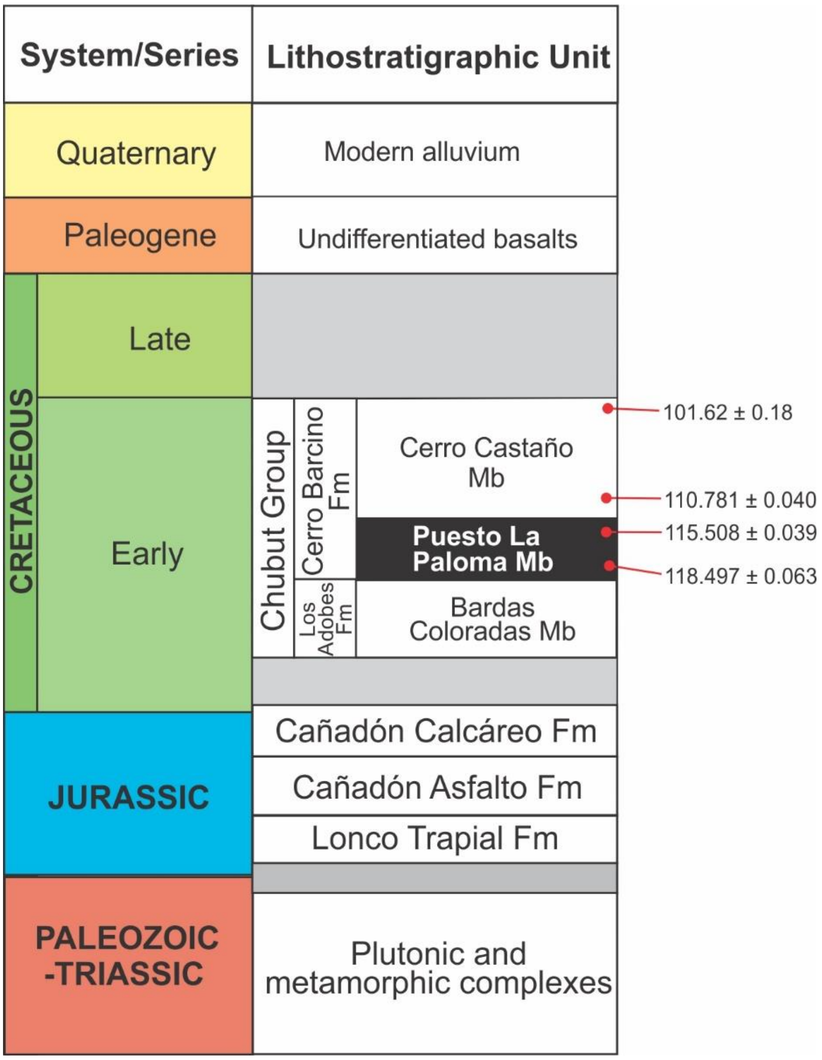

Fig. S1. Stratigraphy of the Somuncurá-Cañadón Asfalto basin at Los Chivos Hill area (modified from Allard et al.[1]) and available radioisotopic ages for the analyzed sequence and overlying stratigraphic unit[2-4]. Basin basement is composed of Paleozoic to Triassic igneous and metamorphic complexes[5]. The Jurassic rocks include the volcano-sedimentary sequence named Lonco Trapial Formation[6], and lacustrine successions with fluvial, alluvial and volcanic influence recognized as Cañadón Asfalto and Cañadón Calcáreo formations[7]. The overlying Cretaceous Chubut Group[8] includes a siliciclastic lower section (Los Adobes Formation) and a pyroclastic-rich upper section (Cerro Barcino Formation). At the study area, the Chubut Group is represented by the fluvial succession of the Bardas Coloradas Member of to the Los Adobes Formation[3,9]; and the pyroclastic-rich alluvial successions of the Puesto La Paloma and Cerro Castaño members of the Cerro Barcino Formation[3,10]. The local sequence is capped by Paleogene mafic volcanic rocks composing lava flows, a neck and several associated dikes[11].

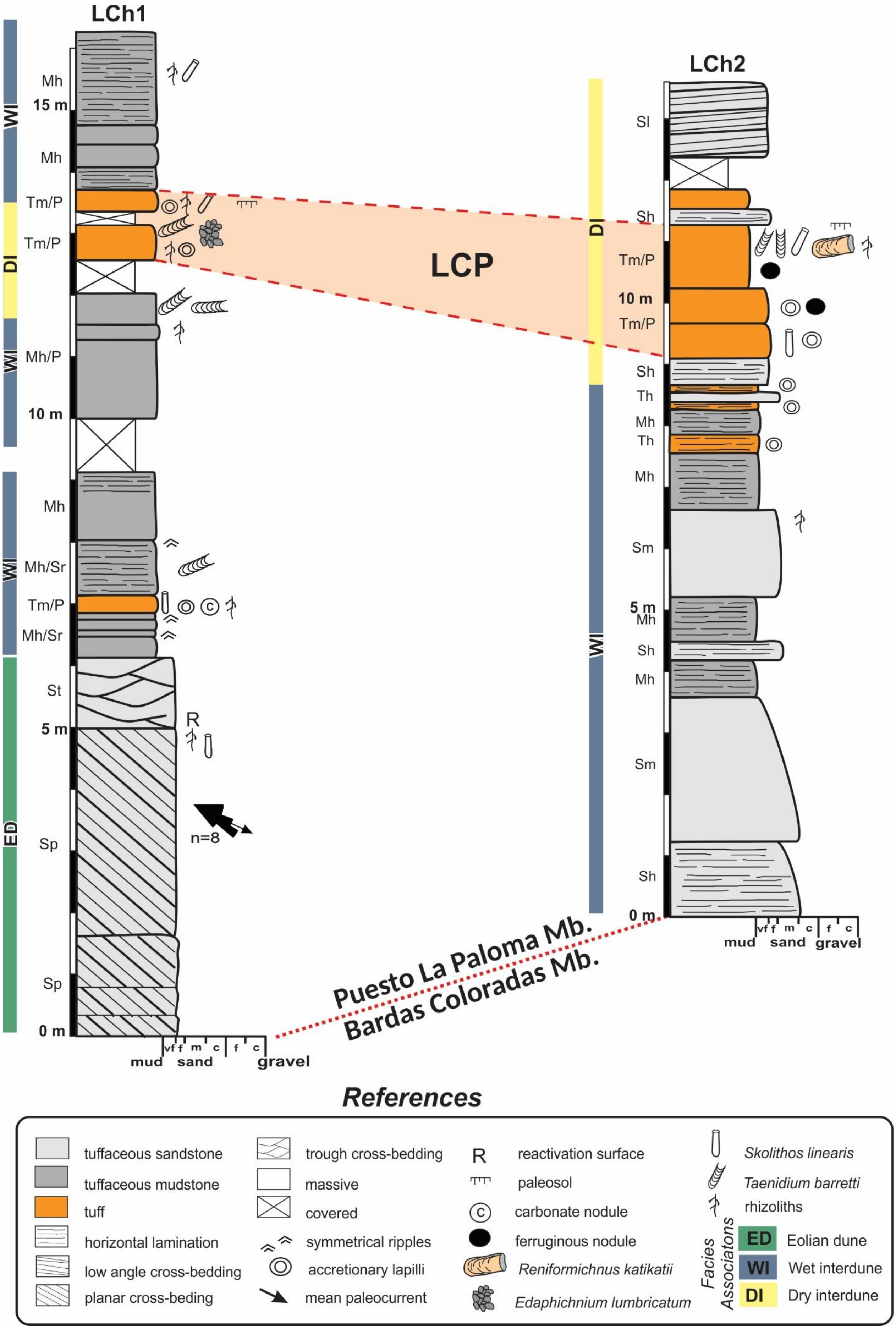
LCh1
LCh2
LCP
Puesto La Paloma Mb.
Bardas Coloradas Mb.
Mh
15 m
Mh
Tm/P
Tm/P
Mh/P
10 m
Mh
Mh/Sr
Tm/P
Mh/Sr
St
5 m
Sp
Sp
0 m
R
n=8
mud sand gravel
vf f m c f c
Sl
Sh
Tm/P
10 m
Tm/P
Sh
Th
Mh
Th
Mh
Sm
5 m
Mh
Sh
Mh
Sm
Sh
0 m
WI
DI
ED
References
tuffaceous sandstone
tuffaceous mudstone
tuff
horizontal lamination
low angle cross-bedding
planar cross-beding
trough cross-bedding
massive
covered
symmetrical ripples
accretionary lapilli
mean paleocurrent
reactivation surface
paleosol
carbonate nodule
ferruginous nodule
Reniformichnus katikatii
Edaphichnium lumbricatum
Skolithos linearis
Taenidium barretti
rhizoliths
Facies Associatons
ED Eolian dune
WI Wet interdune
DI Dry interdune

Fig. S2. Detailed sedimentary logs of the lower Puesto La Paloma Member at sites LCh1 (43° 13' 9.78” S; 68° 50' 36.18” W) and LCh2 (43° 12' 48.56” S; 68° 50' 42.91” W). These sections illustrate the local variability of the lower part of the sequence at the study area. Letters on the left side of logs represent the code of sedimentary facies used (Table S1). The pedogenized ash-fall strata bearing *Reniformichnus katikatii* (in LCh2), which belong to dry interdune facies association, is highlighted. See descriptions and interpretation of facies and facies associations in Table S1.

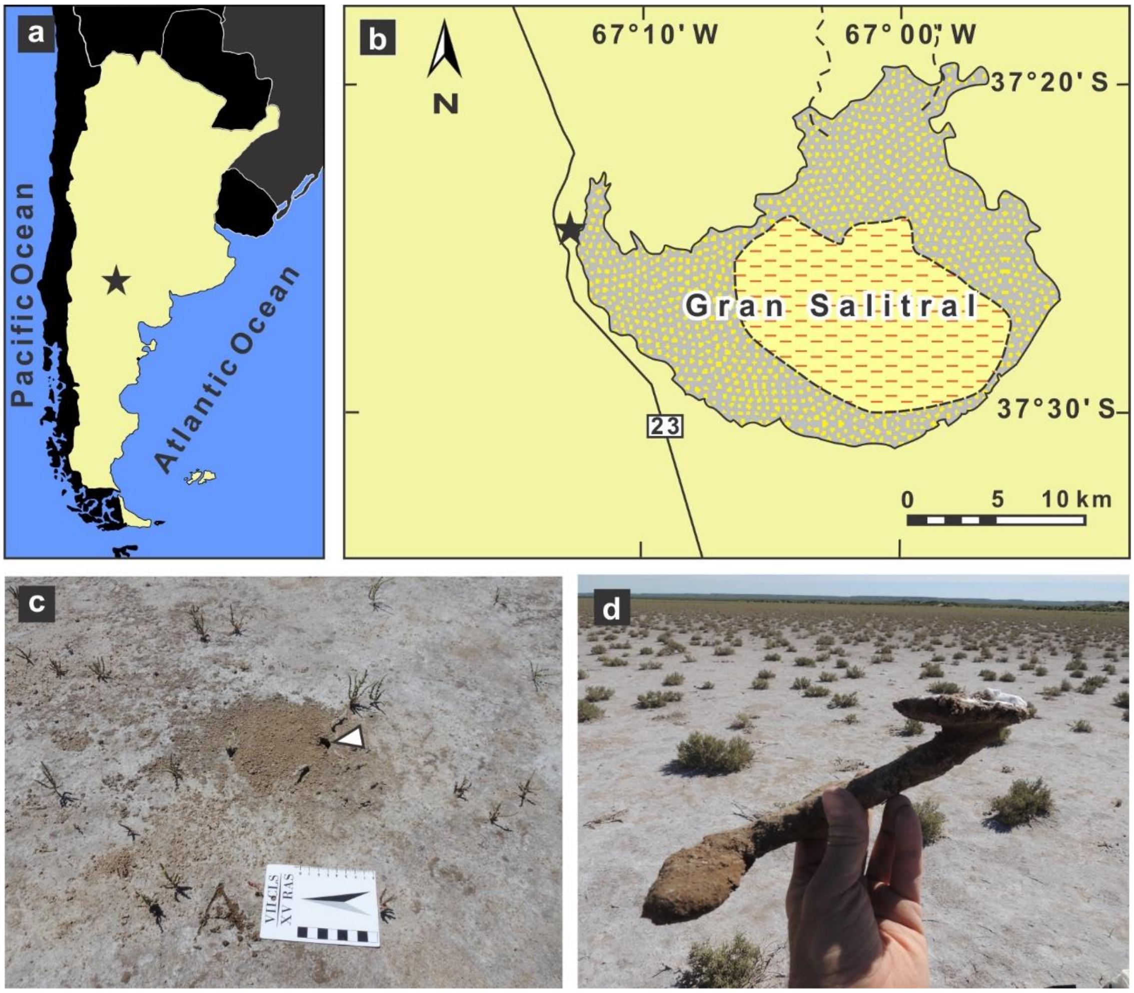

Fig. S3. Location of the site of *Liolaemus* sp. burrow casts. **(a-b)** Puesto La Porfía locality in the center of Argentina (southwestern La Pampa province). GPS coordinates: 37° 24’ 26.50” S, 67° 12’ 29.85” W. **(c)** Surface of saline sandflat with burrow entrance (arrowed) before casting. **(d)** *Liolaemus* sp. burrow cast immediately after excavation (forefront) and vegetated saline sandflat (background).

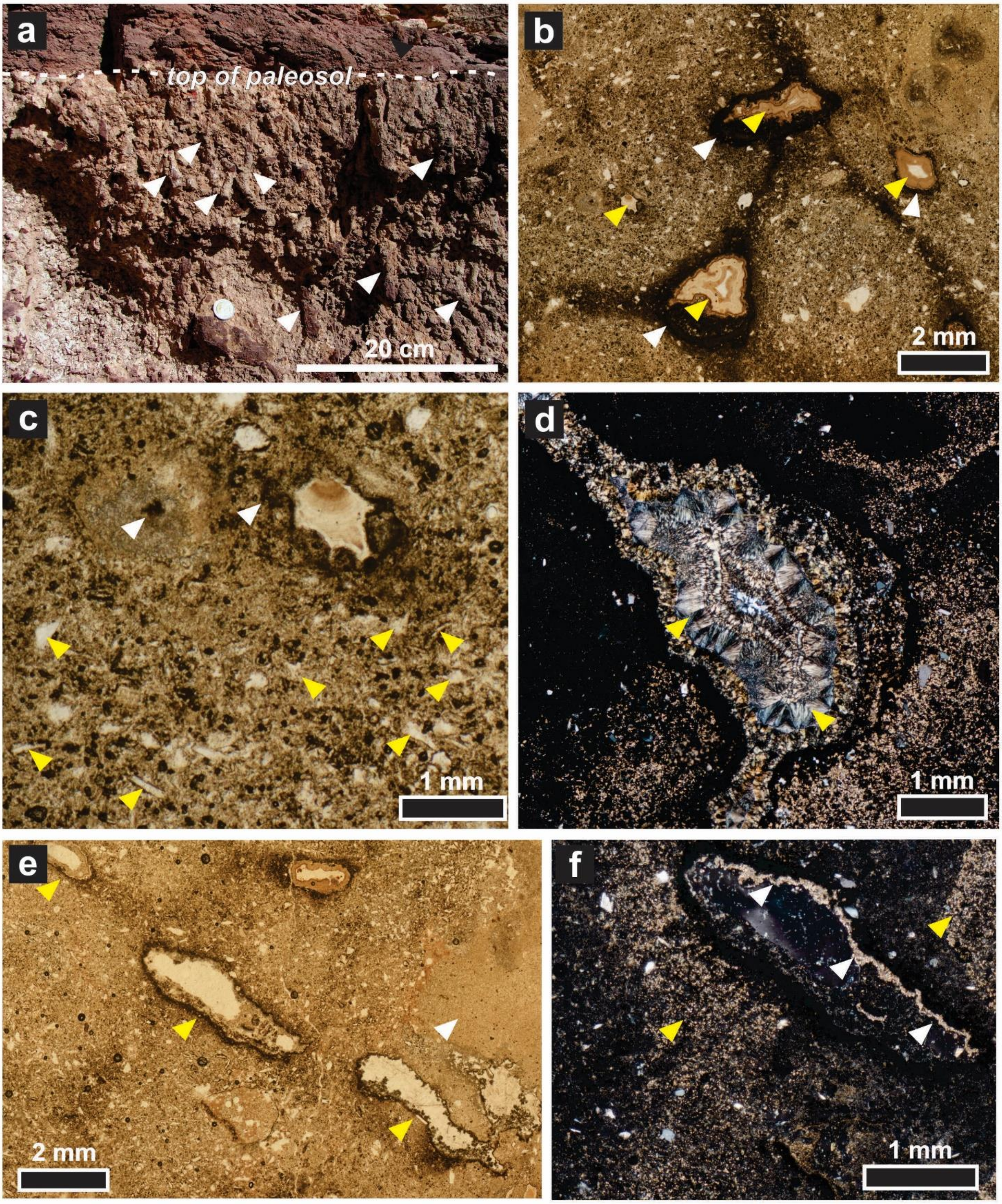

Fig. S4. Paleosol description. **(a)** Field photography of the upper part of the Los Chivos paleosol. This paleosol contains three horizons having transitional boundaries. The upper horizon is a 0.75 m thick, light pinkish white (5YR 9/1) massive fine-grained tuff with millimetric Fe-Mn nodules. It is characterized by the presence of *Reniformichnus katikatii*, vertical meniscate or massive invertebrate burrows (white arrows in **a**) and locally abundant subvertical rhizholiths. The middle horizon is a 0.60 m thick fine-grained tuff with a coarse granular structure, pinkish white (5YR 9/1) to white (5YR 8/1) in color, showing diffuse parallel lamination. The lower horizon is a 110 cm thick, light grey (5YR7/1) massive very fine-grained tuff also showing coarse structure. Thin sections of the paleosol **(b-f)** show an apedal microstructure **(b)** with low porosity (<5%). The grain size of the

coarse fraction (almost 20%) ranges from silt to fine-grained sand, and is mostly composed of glass shards (yellow arrows in **c**), with subordinate tabular plagioclase, potassium feldspar and quartz. The fine fraction is composed of light yellow and brown clays with scarce opaque material (white arrows in **b**, **c** and **e**). The groundmass is open porphyric, with weak development of speckled micrite (yellow arrows in **f**) and an undifferentiated b-fabric. Porosity is randomly distributed (simple packing voids and channels) and microstructure is mostly massive to locally vuggy (yellow arrows in **b** and **e**). The identified microscopic pedofeatures are weakly developed clay and calcite coatings (white arrows in **f**) and impregnative Fe-Mn oxide hypocoatings (white arrows in **b**), dense complete filling of vughs and channels with fibrous chalcedony (yellow arrows in **d**) and matrix. Microphotographs **b**, **c** and **e** taken under transmitted, plane-polarized light. Microphotographs **d** and **f** taken under transmitted, cross-polarized light.

**Facies association**	**Description**	**Interpretation**
Eolian dune	Fine-grained, well-sorted tuffaceous sandstone with sheet-like geometry (6 m thick), good lateral continuity with tabular-planar and trough cross-bedding (facies TSp, TSt). Foreset dip angles are up to 25° and exhibits occasional laminae with inverse grading. Upper part massive with rhizoliths and *Skolithos linearis*. Paleocurrent data with low scattering toward the SE.	Migration of 2D and 3D eolian dunes by grain fall and grain flow processes. The 3D bedforms were probably superimposed on the windward slope or located near the leeward foot of larger 2D dunes. The unimodal pattern of paleocurrent is compatible with deposition on transverse dunes. Upper massive part represents stabilization of the dunes.
Wet interdune	Two- to three-meter-thick tabular bodies with non-erosive bases and a lateral continuity exceeding 100 m, composed of laminated tuffaceous mudstone, massive tuffs with accretionary lapilli, and well sorted medium to fine-grained rippled, massive or laminated tuffaceous sandstone (facies TMh, Tm, TSr, TSm, TSh). Occasional root traces, *Skolithos linearis*, and *Taenidium barretti*.	Flat area between eolian dunes where sediments arrived via fluvial processes or primary pyroclastic influx. Occasional ponding of waters. Levels with root traces suggest subaerial exposure and soil-forming processes.
Dry interdune / extradune?	Up to 5 m thick, sheet-like body with non-erosive bottom composed of massive very-well sorted fine-grained tuff with accretionary lapilli and the Los Chivos paleosol (facies Tm and P). Intercalations of well-sorted fine-grained tuffaceous sandstone with horizontal and low-angle cross-bedding (facies TSh and TSl). *Reniformichnus katikatii*, *Skolithos linearis*, *Edaphichnium lumbricatum*, *and Taenidium barreti*.	Subaerial settling of suspended volcanic ash and wind reworking resulting in plane bed and ripple migration. Ash fall deposits were subject to plant rooting, colonization by animals and soil development.

Table S1. Description and interpretation of facies associations recognized in the sedimentary logs of the La Paloma Member at the studied localities (compare Fig. S2).

Category	Ichnotaxobase	*Reniformichnus katikatii* (type material)[12]	*Reniformichnus australis*[13]	*Reniformichnus katikatii* (this study)
Generic	Overall architecture	Simple ramp (< 30°), slight curvature in plan view	Single ramp (entrance up to 70° passing to < 20° to horizontal)	Single ramp (<20° to subhorizontal), slight curvature in plan view
	Cross-sectional shape (RDI)	0.48 ± 0.04 (n = 6)*	0.43	0.53 ± 0.02 (n = 20)*
	Burrow filling	Massive	Massive to laminated	Massive
	Presence of bilobed bottom	yes	Inconsistently bilobed	yes
	Presence of chamber	no	no	no
Specific	Primary surface ornamentation	Claw traces	Absent to subtly preserved claw traces	Claw traces
	Pattern of primary surface ornamentation	Uncommon sets of 2 or 3 traces on burrow cast roof, longer in laterals, traces sets forming a rhomboid pattern in bottom	Crescentic traces on ceiling, none on bottom	Claw traces composing an arcuate pattern on roof and laterals, and low angle chevron pattern on bottom
	Number of claw traces in set	2 or 3	no	3
	Average width of claw trace set (mm)	16.23	no	Roof: 8.89 ± 0.55 mm (n = 26)*, Bottom: 8.48 ± 0.47 mm (n = 7)*
	Secondary surface ornamentation	none	none	Sparse subcircular millimeter-wide burrows
	Ramp Dh (mm)	120.8 ± 8.4 mm (n= 5)*	87.0 mm	63.34 ± 2.07 (n = 27)*

Table S2. Comparison of described burrows with the proposed ichnospecies of the ichnogenus *Reniformichnus*. The described burrow casts share all the ichnotaxobases (i.e., morphological feature of a trace fossil that is considered valid for ichnotaxonomy[14]) of the ichnogenus *Reniformichnus*, and also the specific ichnotaxobases of the ichnospecies *R. katikatii*. **RDI** (Relative diameter index)[15]= Ratio of vertical to horizontal diameter (RDI = Dv/Dh) taken at the same point in the cast. **Dv** (vertical diameter) = Maximum vertical distance between the roof and bottom of the ramp or chamber, taken perpendicular to the burrow axis. **Dh** (horizontal diameter) = Maximum horizontal distance between the sides of a ramp or chamber taken perpendicular to the burrow axis. **Primary surface ornamentation**[15,16] **=** Sets of curved parallel ridges (in the cast) or grooves (in the burrow margin) representing excavation by the producer that are interpreted as claw or tooth traces (bioglyphs[17]). **Secondary surface ornamentation**[15,16] **=** cylindrical protuberances that are considerably smaller than the hosting burrow, and cannot be linked to digging by the tetrapod producer. They are essentially

interpreted as invertebrate burrows and/or rhizoliths. (*) indicates average values ± standard error and number of observations.

Age		Squamata	Sphenodontia	Mammalia
Late Cretaceous	Maastrichtian	- -	- -	- -
	Campanian	Undetermined Scincomorpha[18] Partial dentary 10 mm long (inferred skull width ~ 23 mm). Body mass: Inferred ~ 500 g[18,19]. Habit: unknown	*Kawasphenodon expectatus*[20] Body mass: lower jaw 110 mm (largest Sphenodontian). Habit: aquatic?[20]	
	Coniacian	- -	- -	- -
	Santonian	*Paleochelco occultato*[21] Body mass: Partial skull 10 mm wide. Habit: unknown.	- -	- -
	Turonian	- -	*Patagosphenos watuku*[22] Body mass: fragmentary dentary is 18 mm. Habit: fossorial adaptations.	- -
	Cenomanian	?Iguanidae[23] Frontal remain 2 mm wide. Body mass: unknown. Habit: unknown	*Tika giacchinoi* [24] Skull length 39-42 mm Body mass[25]: 47-64 g. Habit: not fossorial. Insectivore/carnivore?. *Kaikaifilusaurus avelasi*[26] Skull length: 120 mm, snout-vent length: 550 mm. Body mass[25] >> 820 g. Habit: herbivorous.	*Cronopio dentiacutus*[27] Body mass: skull length is 27 mm, body mass[28]: 27 g. Habit: unknown.
Early Cretaceous	Albian	- -	- -	- -
	Aptian	- -	*Kaikaifilusaurus (Priosphenodon) minimus*[29] Small size. Subadult skull length ~ 20 mm. Body mass[25]: 7 g. Habit: fossorial, herbivorous, gregarious.	*Vincelestes nuequenianus*[30] Body mass[31]: 619 to 1228 g. Habit: scansorial-arboreal.
	Barremian	- -	- -	

Table S3. Compilation of potential producers of *Reniformichnus katikatii* from the Los Chivos paleosol, ordered by age, body mass and habit. See discussion in text.

1 Allard, J. O. *et al.* in *Geología y Recursos Naturales de la Provincia del Chubut. Relatorio del 21° Congreso Geológico Argentino* (ed R.E. Giacosa) 187-265. (2022).

2 Carballido, J. L. *et al.* A new giant titanosaur sheds light on body mass evolution among sauropod dinosaurs. *Proceedings of the Royal Society B: Biological Sciences* **284**, 20171219, doi:10.1098/rspb.2017.1219 (2017).

3 Krause, J. M. *et al.* High-resolution chronostratigraphy of the Cerro Barcino Formation (Patagonia): Paleobiologic implications for the mid-cretaceous dinosaur-rich fauna of South America. *Gondwana Research* **80**, 33-49, doi:https://doi.org/10.1016/j.gr.2019.10.005 (2020).
4 Suárez, M., Márquez, M., De La Cruz, R., Navarrete, C. & Fanning, M. Cenomanian-? early Turonian minimum age of the Chubut Group, Argentina: SHRIMP U–Pb geochronology. *Journal of South American Earth Sciences* **50**, 67-74, doi:https://doi.org/10.1016/j.jsames.2013.10.008 (2014).
5 Figari, E. G., Scasso, R. A., Cúneo, R. N. & Escapa, I. Estratigrafía y evolución geológica de la cuenca de Cañadón Asfalto, provincia del Chubut, Argentina. *Latin American Journal of Sedimentology and Basin Analysis* **22**, 135-169 (2015).
6 Zaffarana, C. B. *et al.* in *Geología y Recursos Naturales de la Provincia del Chubut. Relatorio del 21° Congreso Geológico Argentino* (ed R.E. Giacosa) 344-363 (2022).
7 Cúneo, R. *et al.* High-precision U–Pb geochronology and a new chronostratigraphy for the Cañadón Asfalto Basin, Chubut, central Patagonia: Implications for terrestrial faunal and floral evolution in Jurassic. *Gondwana Research* **24**, 1267-1275, doi:https://doi.org/10.1016/j.gr.2013.01.010 (2013).
8 Codignotto, J., Nullo, F., Panza, J. & Proserpio, C. in *7° Congreso Geológico Argentino Actas.* 471-480.
9 Villegas, P. M., Visconti, G. & Umazano, A. M. in *14° Reunión Argentina de Sedimentología.* 295-296 (Asociación Argentina de Sedimentología).
10 Umazano, A. M. *et al.* Changing fluvial styles in volcaniclastic successions: A Cretaceous example from the Cerro Barcino Formation, Patagonia. *Journal of South American Earth Sciences* **77**, 185-205, doi:https://doi.org/10.1016/j.jsames.2017.05.005 (2017).
11 Proserpio, C. A. Descripción geológica de la Hoja 44e, Valle General Racedo, Provincia del Chubut. *Dirección Nacional de Minería y Geología, Boletín* **201**, 1-108 (1987).
12 Krummeck, W. D. & Bordy, E. M. *Reniformichnus katikatii* (new ichnogenus and ichnospecies): Continental vertebrate burrows from the Lower Triassic, main Karoo Basin, South Africa. *Ichnos* **25**, 138-149, doi:10.1080/10420940.2017.1292909 (2018).
13 McLoughlin, S. *et al.* Dwelling in the dead zone—Vertebrate burrows immediately succeeding the end-Permian extinction event in Australia. *PALAIOS* **35**, 342-357, doi:10.2110/palo.2020.007 (2020).
14 Bromley, R. G. *Trace fossils. Biology and taphonomy*. (Unwin Hyman, 1990).
15 Cardonatto, M. C. & Melchor, R. N. Recognition of fossil nebkha deposits: Clues from neoichnology and sedimentology. *Palaios* **35**, 277–291, doi:10.2110/palo.2020.024 (2020).
16 Cardonatto, M. C. & Melchor, R. N. Environmental influence on burrow system features of a colonial and fossorial rodent. Implications for interpreting fossil tetrapod burrows. *Palaios* **36**, 225-245, doi:10.2110/palo.2020.065 (2021).
17 Bromley, R. G. *Trace Fossils: Biology, taphonomy and applications*. second edn, (Chapman & Hall, 1996).
18 Brizuela, S. & Albino, A. A Scincomorpha lizard from the Campanian of Patagonia. *Cretaceous Research* **32**, 781-785, doi:https://doi.org/10.1016/j.cretres.2011.05.006 (2011).
19 Pough, F. H. Lizard energetics and diet. *Ecology* **54**, 837-844, doi:https://doi.org/10.2307/1935678 (1973).
20 Apesteguía, S. A Late Campanian sphenodontid (Reptilia, Diapsida) from northern Patagonia. *Comptes Rendus - Palevol* **4**, 663-669, doi:10.1016/j.crpv.2005.06.003 (2005).
21 Martinelli, A. G., Agnolín, F. L. & Ezcurra, M. D. Unexpected new lizard from the Late Cretaceous of southern South America sheds light on Gondwanan squamate diversity. *Rev Mus Arg Cs Nat* **23**, 57-80., doi:http://dx.doi.org/10.22179/revmacn.23.716 (2021).
22 Gentil, A. R., Agnolin, F. L., Garcia Marsà, J. A., Motta, M. J. & Novas, F. E. Bridging the gap: Sphenodont remains from the Turonian (Upper Cretaceous) of Patagonia. Palaeobiological inferences. *Cretaceous Research* **98**, 72-83, doi:https://doi.org/10.1016/j.cretres.2019.01.016 (2019).
23 Apesteguía, S., Agnolin, F. L. & Lio, G. L. An early Late Cretaceous lizard from Patagonia, Argentina. *Comptes Rendus - Palevol* **4**, 311-315, doi:10.1016/j.crpv.2005.03.003 (2005).

24 Apesteguía, S., Garberoglio, F. F. & Gómez, R. O. Earliest Tuatara relative (Lepidosauria: Sphenodontinae) from southern continents. *Ameghiniana* **58**, 416-441, doi:10.5710/AMGH.13.07.2021.3442 (2021).
25 Herrel, A., Schaerlaeken, V., Moravec, J. & Ross, C. F. Sexual shape dimorphism in tuatara. *Copeia* **2009**, 727-731 (2009).
26 Apesteguía, S. *Esfenodontes (Reptilia: Lepidosauria) del Cretácico Superior de Patagonia* PhD thesis, Universidad Nacional de La Plata, (2008).
27 Rougier, G. W., Apesteguía, S. & Gaetano, L. C. Highly specialized mammalian skulls from the Late Cretaceous of South America. *Nature* **479**, 98-102, doi:10.1038/nature10591 (2011).
28 Luo, Z.-X., Crompton, A. W. & Sun, A.-L. A new mammaliaform from the Early Jurassic and evolution of mammalian characteristics. *Science* **292**, 1535-1540, doi:10.1126/science.1058476 (2001).
29 Apesteguía, S. & Carballido, J. L. A new eilenodontine (Lepidosauria, Sphenodontidae) from the Lower Cretaceous of central Patagonia. *J Vertebr Paleontol* **34**, 303-317, doi:10.1080/02724634.2013.803974 (2014).
30 Rougier, G. W. *Vincelestes neuquenianus Bonaparte (Mammalia, Theria), un primitivo mamífero del Cretácico Inferior de la Cuenca Neuquina* PhD thesis, Universidad de Buenos Aires, (1993).
31 Macrini, T. E., Rougier, G. W. & Rowe, T. Description of a cranial endocast from the fossil mammal *Vincelestes neuquenianus* (Theriiformes) and its relevance to the evolution of endocranial characters in therians. *The Anatomical Record* **290**, 875-892, doi:https://doi.org/10.1002/ar.20551 (2007).
